# Two Key Amino Acids Variant of α-l-arabinofuranosidase from *Bacillus subtilis* Str. 168 with Altered Activity for Selective Conversion Ginsenoside Rc to Rd

**DOI:** 10.3390/molecules26061733

**Published:** 2021-03-19

**Authors:** Ru Zhang, Shi Quan Tan, Bian Ling Zhang, Zi Yu Guo, Liang Yu Tian, Pei Weng, Zhi Yong Luo

**Affiliations:** 1 College of Materials and Chemical Engineering, Hunan Institute of Engineering, Xiangtan 411104, China; tanshiquan11@163.com (S.Q.T.); blzhang369@163.com (B.L.Z.); g13367365606@163.com (Z.Y.G.); tianliangyu2021@163.com (L.Y.T.); wp1475963@163.com (P.W.); 2 Hunan International Joint Laboratory of Animal Intestinal Ecology and Health, Laboratory of Animal Nutrition and Human Health, College of Life Sciences, Hunan Normal University, Changsha 410081, China; 3 Molecular Biology Research Center, School of Life Sciences, Central South University, Changsha 410078, China; luo_zhiyong@hotmail.com

**Keywords:** *Bacilus subtilis*, α-l-arabinofuranosidase, ginsenoside Rc, biotransformation, ginsenoside Rd, site-directed mutagenesis, molecular docking

## Abstract

α-l-arabinofuranosidase is a subfamily of glycosidases involved in the hydrolysis of l-arabinofuranosidic bonds, especially in those of the terminal non-reducing arabinofuranosyl residues of glycosides, from which efficient glycoside hydrolases can be screened for the transformation of ginsenosides. In this study, the ginsenoside Rc-hydrolyzing α-l-arabinofuranosidase gene, *BsAbfA,* was cloned from *Bacilus subtilis,* and its codons were optimized for efficient expression in *E. coli* BL21 (DE3). The recombinant protein BsAbfA fused with an N-terminal His-tag was overexpressed and purified, and then subjected to enzymatic characterization. Site-directed mutagenesis of BsAbfA was performed to verify the catalytic site, and the molecular mechanism of BsAbfA catalyzing ginsenoside Rc was analyzed by molecular docking, using the homology model of sequence alignment with other β-glycosidases. The results show that the purified BsAbfA had a specific activity of 32.6 U/mg. Under optimal conditions (pH 5, 40 °C), the kinetic parameters *K_m_* of BsAbfA for *p*NP-α-Araf and ginsenoside Rc were 0.6 mM and 0.4 mM, while the *K_cat_/K_m_* were 181.5 s^−1^ mM^−1^ and 197.8 s^−1^ mM^−1^, respectively. More than 90% of ginsenoside Rc could be transformed by 12 U/mL purified BsAbfA at 40 °C and pH 5 in 24 h. The results of molecular docking and site-directed mutagenesis suggested that the E173 and E292 variants for BsAbfA are important in recognizing ginsenoside Rc effectively, and to make it enter the active pocket to hydrolyze the outer arabinofuranosyl moieties at C_20_ position. These remarkable properties and the catalytic mechanism of BsAbfA provide a good alternative for the effective biotransformation of the major ginsenoside Rc into Rd.

## 1. Introduction

Ginseng, as a famous traditional herbal medicine, has been used to cure diseases and promote health in East Asia for thousands of years. In recent decades, the medicinal value of ginseng has been recognized worldwide [1,2]. A large number of studies have shown that ginsenosides play pivotal pharmacological and therapeutic roles [3,4,5]. Among over 100 ginsenosides isolated and identified from ginseng, five major ginsenosides, viz., Rb_1_, Rb_2_, Rc, Re, and Rg_1_, account for more than 80% of all ginsenosides [6,7,8]. Ginsenoside Rd has been proved to have unique pharmacological activities, such as reducing the proliferation and migration of glioblastoma cells [9], attenuating breast cancer metastasis [10], stimulating the proliferation of endogenous stem cells [11], improving the blood–brain barrier in ischemic stroke [12], attenuating mitochondrial dysfunction, and sequential apoptosis after transient focal ischemia [13]. Due to the value of ginsenoside Rd in medicinal applications, and as a promising medicine candidate, its transformation from other ginsenosides has been investigated. However, the content of ginsenoside Rd in nature is much less than that of the major ginsenosides [5,7], which makes it particularly difficult to obtain ginsenoside Rd from ginseng and other plants. Some researchers have tried to obtain ginsenoside Rd by chemical synthesis, but found it difficult to succeed, because of its complex structure [14].

**Figure 1:**
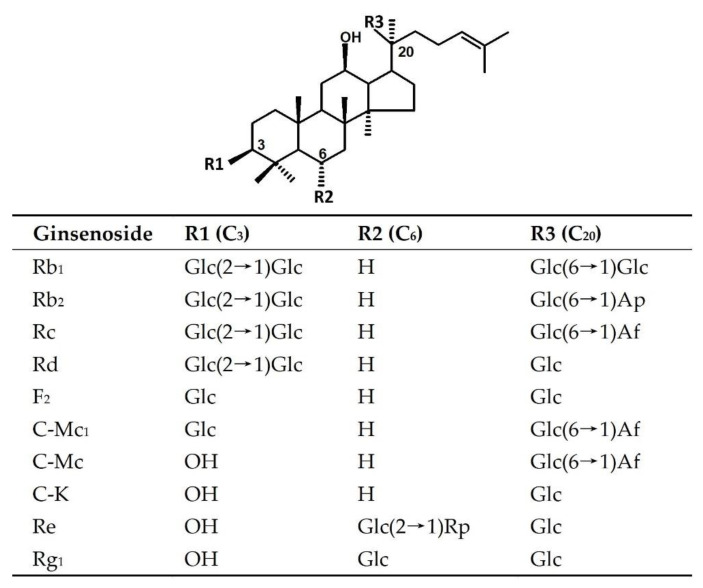
Chemical structure of ginsenoside Rb_1_, Rb_2_, Rc, Rd, Re, Rg_1_, F_2_, C-K, C-Mc, and C-Mc_1_. Glc, Ap, Af, and Rp are abbreviations of glucopyranosyl, arabinopyranosyl, arabinofuranosyl, and rhamnopyranosyl, respectively.

Ginsenosides are tetracyclic triterpenoids with similar structures (Figure 1). Most ginsenosides belong to the protopanaxadiol type, the main difference lies in the variety and quantity of C_3_ and C_20_ sugar groups. Ginsenoside Rd is structurally similar to Rb_1,_ Rb_2_, Rb_3_, and Rc, but lacks one outer glycoside moiety at position C_20_ [8]. Therefore, it is feasible to obtain ginsenoside Rd from Rb_1,_ Rb_2_, Rb_3_, and Rc by hydrolyzing the outer monosaccharide residue (i.e., arabinopyranose or arabinofuranose moieties) using a specific glycosidase, such as β-glucosidase, α-l-arabinopyranosidase, or α-l-arabinofuranosidase [15,16,17,18]. In all the major ginsenosides, the content of Rc accounts for about 20% of the total ginsenosides [7,15]. Therefore, it can serve as an important substrate for ginsenoside Rd production. Hence, it is essential to screen enzymes and/or reagents that can cleave the arabinofuranose at the C_20_ position of ginsenoside Rc with high activity and specificity. At present, physical and chemical methods have been developed to obtain ginsenoside Rd from other major ginsenosides, but the efficiency and specificity are not ideal [19]. Glycosidases are widely distributed in almost all organisms, which hydrolyze glycosidic bonds in various glycosides by endo- or exo-digestion. Compared to the known physical and chemical methods, the preparation of rare ginsenosides by hydrolysis of glycosidic bonds using glycosidase has advantages, such as high selectivity, mild reaction condition, and environmental friendliness [20]. α-l-arabinofuranosidase (EC 3.2.1.55) is a subfamily of glycosidases commonly involved in the hydrolysis of l-arabinofuranosidic bonds, especially in the hydrolysis of terminal non-reducing arabinofuranosyl residues from different oligosaccharides and polysaccharides [21,22]. Some α-l-arabinofuranosidases from *Bifidobacterium breve* K-110 [23], *Rhodanobacter ginsenosidimutans* Gsoil 3054T [15], *Caldicellulosiruptor saccharolyticus* [24], *Sulfolobus solfataricus* [25], *Thermotoga thermarum* DSM5069 [26], and *Geobacillus caldoxylolyticus* TK4 [27] have been proved to possess the ability of transforming ginsenoside Rc into ginsenoside Rd. Although there are several α-l-arabinofuranosidases from different species that are known to have the potential of preparing ginsenoside Rd, there have been no reports on the scaled-up production of ginsenoside Rd using α-l-arabinofuranosidase. This is plausibly due to limitations such as low activity and poor extraction efficiency for natural enzymes, as well as low expression level, poor substrate specificity, and low transformation ability for recombinant enzymes. Moreover, the mechanism of ginsenoside hydrolysis by glycosidase is unclear, enormously hindering further modification and optimization of the enzyme. Therefore, it is urgent to investigate the efficient and specific hydrolysis of ginsenoside Rc using α-l-arabinofuranosidase.

**Figure 2:**
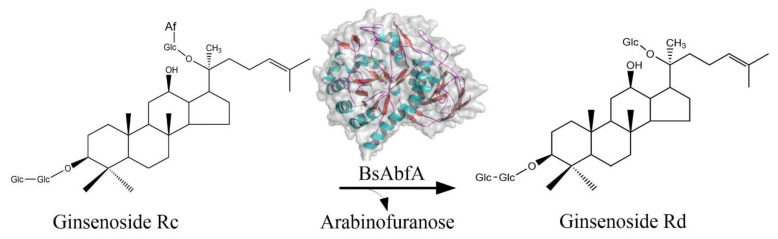
Proposed biotransformation pathway of ginsenoside Rc into Rd by BsAbfA.

In this paper, the ginsenoside Rc-hydrolyzing α-l-arabinofuranosidase gene *BsAbfA* was cloned from *B. subtilis* and its codons were optimized for efficient expression in *E. coli*. The optimized recombinant protein, BsAbfA, fused with an N-terminal His-tag was overexpressed and purified, and then subjected to enzymatical characterization. The catalytic efficiency of recombinant BsAbfA on biotransformation of the major ginsenoside Rc to Rd was investigated (Figure 2). We aimed to explore more α-l-arabinofuranosidases for efficient enzymatic production of ginsenoside Rd. We performed a site-directed mutagenesis of BsAbfA to verify the catalytic site. Furthermore, we analyzed the molecular mechanism of BsAbfA catalysis on ginsenoside Rc by molecular docking using the homology model of sequence alignment with other β-glycosidases. The E173 and E292 variants of BsAbfA are important for the effective recognition of ginsenoside Rc, leading to its entrance to the active pocket for the hydrolysis of the outer arabinofuranose moieties at C_20_ position. These remarkable properties and catalytic mechanism of BsAbfA provide a good alternative for the effective biotransformation of ginsenoside Rc into Rd.

## 2. Results and Discussion

### 2.1. Cloning of BsAbfA Gene and Sequence Analysis

**Figure 3:**
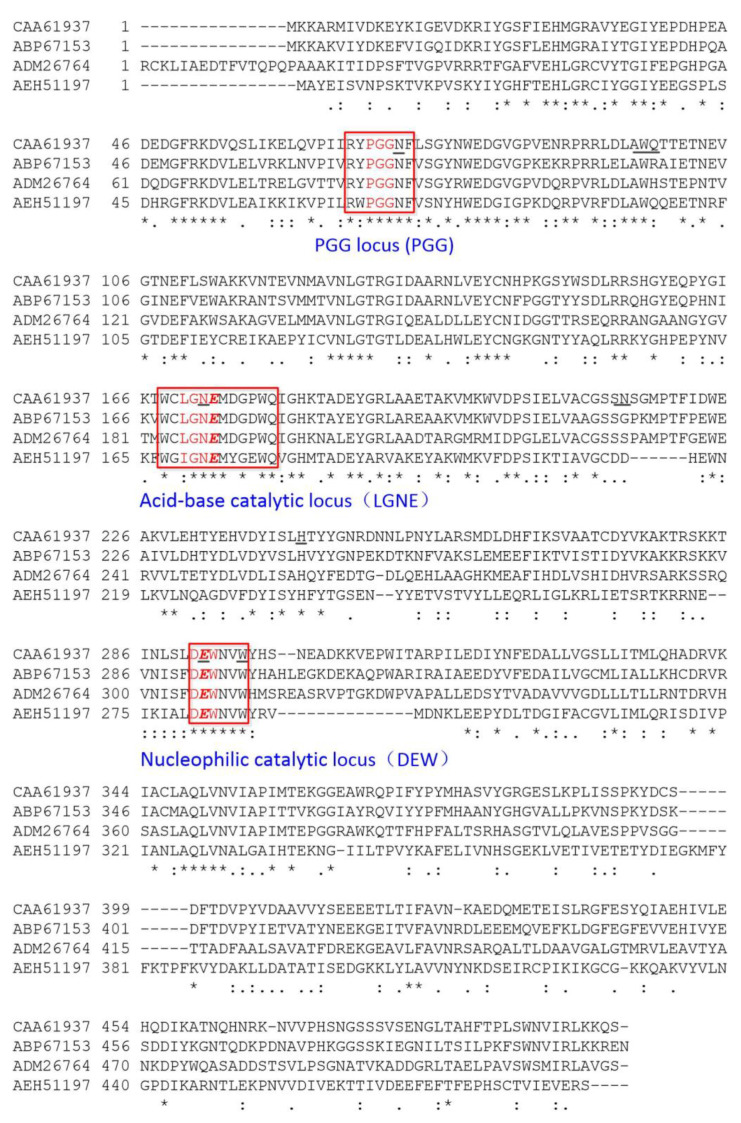
Multiple sequence alignment of BsAbfA with α-l-arabinofuranosidases from several microorganisms: CAA61937 (BsAbfA) from *B. subtilis*, ADM26764 from *R. ginsenosidimutans* Gsoil 3054, ABP67153 from *T. thermarum* DSM5069, and AEH51197 from *Pseudothermotoga thermarum* DSM 5069. The three motifs with conserved RYPGG (residues 67–73), WCLGNEMDGPWQ (residues 168–179), and DEWNVW (residues 291–296) are highlighted in a box. The residues E173 and E292 (Red bold italic letters) are regarded as typical and necessary acid-base and nucleophilic catalytic residues for the hydrolysis of the glycosidic bond.

In the structural characterization of BsAbfA (GenBank accession: AL009126.3, 2938330–2939832) an alignment of the amino acid sequence of BsAbfA with several GH51 α-l-arabinofuranosidases by using ClustalX indicated the sharing of three conserved motifs. Studies have shown that arabinofuranosidases are involved in general acid-base catalysis, and two essential residues are required in the process of cleaving glycosidic bonds. In most arabinofuranosidases conservative aspartic acid and/or glutamic acid residues are available and needed [22,28]. Among the two catalytic residues, one acts as a general acid to provide proton assistance for the departure of glycosidic oxygen, and the other acts as a general base to activate a water molecule and affect the direct replacement at the anomeric center [22]. As shown in Figure 3, the first motif with a conserved RYPGG sequence is regarded as an important motif for stabilizing the structure and confers flexibility to the enzyme [29]. According to sequence similarities with BsAbfA, the motifs of WCLGN***E***MDGPWQ (residues 168–179) and D***E***WNVW (residues 291–296) are highly reserved with the GH51 α-l-arabinofuranosidases, and the residues E173 and E292 (Red bold italic letters) are regarded as typical and necessary acid-base and nucleophilic catalytic residues to hydrolyze the glycosidic bond [22,30,31], respectively. The three conserved motifs of BsAbfA are basically the same as those of AbfA (GenBank accession No. ADM26764) from *R. ginsenosidimutans* Gsoil 3054. This AbfA shows preferential substrate specificity for exo-polyarabinosides or oligoarabinosides, and it only hydrolyzes arabinofuranoside moieties from ginsenoside Rc and its derivatives, and no other sugar groups [15]. Between BsAbfA and the α-l-arabinofuranosidase (GenBank accession No. ABP67153) from *T. thermarum* DSM5069, we found only one residue difference in the three motifs. It is worth noting that they also have a high specific ability to biotransform ginsenoside Rc to Rd through the hydrolysis of the arabinoside bond [26]. Therefore, we speculate that they may share a similar catalytic mechanism.

### 2.2. Identification of Enzymatic Properties of Recombinant BsAbfA

The wild-type BsAbfA gene without any optimization was cloned and fused in pET-28a (+), and the results showed that expression in *E. Coli* BL21 (DE3) was difficult. Therefore, the codons of wild-type BsAbfA were optimized and synthesized for expression efficiency. The optimized and mutated BsAbfAs fused to His-tag were purified using Ni-NTA magnetic agarose beads from *E. Coli* BL21 (DE3), followed by the induction of 0.5 mM IPTG at 20 °C for 16 h. SDS-PAGE analyses revealed that the molecular masses of all the optimized and mutated BsAbfAs were similar to those of those predicted according to amino acid sequences (approximately 60 kDa, data not shown). The results indicated that the mutated BsAbfAs were correctly expressed and folded. The purified BsAbfA, with 32.6 U/mg and using *p*-nitrophenyl-α-l-arabinofuranoside (*p*NP-α-Af) as substrate, displayed a higher activity than most of the characterized α-l-arabinofuranosidases, such as α-l-arabinofuranosidase from *C. saccharolyticus* with 28.2 U/mg [32]. The crude enzyme and purified recombinant α-l-arabinofuranosidase from *Cellulosimicrobium aquatile* Lyp51 showed a lower activity of 2 U/mg and 15 U/mg, respectively [33]. The activity of *T. petrophila* α-l-arabinofuranosidase purified from *E. Coli* BL21 (DE3) toward ginsenoside Rc was only 10.3 U/mg at the optimal condition [20]. The ginsenoside-hydrolyzing α-l-arabinofuranosidase from *R. ginsenosidimutans* Gsoil 3054T was 14.9 U/mg [15]. To investigate the importance of the two key residues (i.e., E173 and E292) for hydrolysis of ginsenoside Rc, each one was independently substituted by an alanine, asparagine, and glutamine. It was found that the E173A or E292A mutants had no specific activity toward ginsenoside Rc and *p*NP-α-Af. Furthermore, the mutants of E173D, E173Q, E292D, and E292Q led to an obvious decrease of activity, and the extent of activity loss was 73.4%, 46.1%, 76.4%, and 56.1%, respectively, compared to that of BsAbfA using ginsenoside Rc as a substrate (Table 1). For investigation of the catalytic mechanism, researchers have attempted to identify the residues of α-l-arabinofuranosidase that are catalytically essential. They found that all enzymes of this superfamily possess conserved glutamates E173 and E292 (numbering for BsAbfA based on the alignment), which are catalytically essential acid-bases and nucleophiles, respectively [22,34].

### 2.3. Temperature and pH Dependence of Recombinant BsAbfA

**Figure 4:**
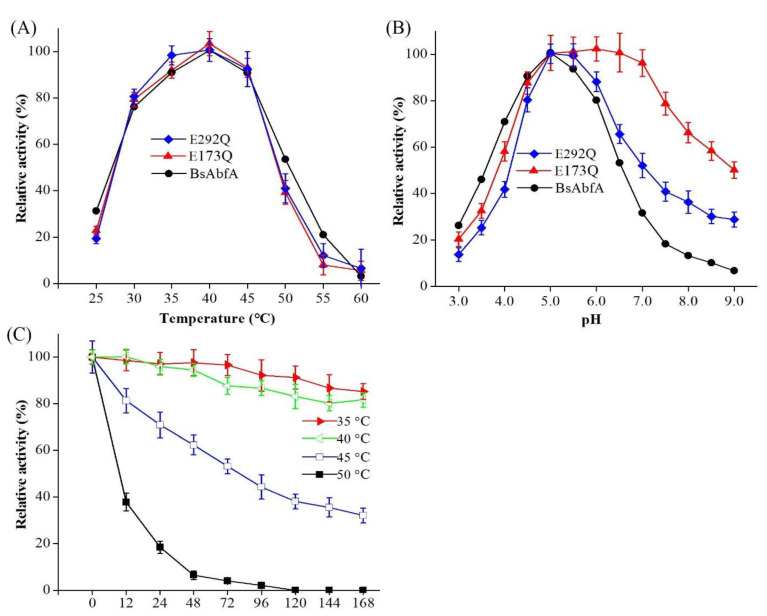
Temperature, pH dependence, and thermal stability of enzymatic activity of BsAbfA. (**A**) Activity measurements catalyzed by BsAbfA, E173Q, and E292Q mutants at 25‒60 °C with pH 5. (**B**) Activity measurements catalyzed by BsAbfA, E173Q, and E292Q mutant at 40 °C; the buffers in a pH range of 3‒9 were citric acid-sodium citrate (50 mM, pH 3‒6), sodium phosphate (100 mM, pH 6‒8), and glycine-NaOH (50 mM, pH 9–10). (**C**) The thermal stabilities were tested at 35, 40, 45, and 50 °C, catalyzed by BsAbfA at pH 5. The maximum relative activity of each enzyme was defined as 100% using *p*NP-α-Af as substrate.

To investigate the temperature and pH dependence of BsAbfA, the enzymic activity of BsAbfA and isosteric mutants E173Q and E292Q were determined using *p*NP-α-Af as substrate at different temperatures and pH environments. As disclosed in Figure 4A, the recombinant BsAbfA and mutants were active at 30–50 °C and relatively stable at 25–45 °C. The optimal temperature was 40 °C. When the temperature was higher than 45 °C, the activity decreased sharply. The activity loss for all the tested enzymes was more than 60%, 80%, and 90% at 50, 55, and 60 °C, respectively. It is known that many glycosidases exhibit optimal activity at mild temperature conditions. For example, the optimal temperatures of the glycosidase from *Leuconostoc mesenteroides* DC102 [35], β-glucosidase from *Lactobacillus brevis* [36], β-d-xylosidase, α-l-arabinopyranosidase, and α-l-arabinofuranosidase from *Bifidobacterium breve* K-110 [23,37], α-l-arabinofuranosidase from *Leuconostoc* sp. 22-3 [16], as well as those of soil deuteromycete *Penicillium funiculosum* [16] and *Cellulosimicrobium aquatile* Lyp51 [33] are 30–45 °C for ginsenosides biotransformation.

As shown in Figure 4B, the pH dependent curves of the activity of BsAbfA and E292Q mutants display an increase of pH sensitivity, while E173Q is characterized by a marked insensitivity to pH. Higher activities were obtained at pH 4.5‒6 for BsAbfA and E292Q mutant. In 50 mM citric acid/sodium citrate buffer, the BsAbfA and E292Q mutants showed maximal activity at pH 5, and activities of above 80% were retained at pH 6. When the pH was above 6 or below 4.5, the enzyme activity decreased significantly. Interestingly, the activity of E173Q mutant remained at a high level with the increase of pH until the pH was higher than 7. The results suggest that the mutation of E173 residue should lead to significant alteration of pH dependence. In this case, the activity of the mutant E173Q is consistent with that of a mutant enzyme that is without catalytic acid-base residue, as reported in the literature [22,38,39]. The main change of the pH dependence curve as a result of E173 mutation supports the view that just like other α-l-arabinofuranosidases, the residue is an acid-base catalyst [38,40,41]. Therefore, it is reasonable to consider that the sites of glutamate residues relative to each other, as well as to the substrate should be critical for efficient catalysis. In this study, no matter if it was *p*NP-α-Af or ginsenoside Rc that was used as substrate, the temperature or pH conditions for maximum enzyme activity stayed the same.

The thermal stability of BsAbfA at pH 5 versus incubation time is depicted in Figure 4C. The thermodynamic parameters confirm that BsAbfA was stable below 40 °C and it had a half-life of 225.7, 196.9, and 74.6 h at 35, 40, and 45 °C, respectively. The enzyme decreased significantly in stability above 50 °C, and the half-life was only 10.8 h. BsAbfA has a half-life comparable to those of characterized α-l-arabinofuranosidase from other species at its optimized temperature. For example, the half-life of α-l-arabinofuranosidase originating from *S. solfataricus* is 30 h at 85 °C [25]. Furthermore, its half-life is higher than some other β-glucosidases from *Fusarium solani* (159 min, 65 °C) [29] and *Alteromonas* sp. L82 (21 min, 40 °C) [42]. It should be emphasized that during the 3 months storage of the BsAbfA (at 4 °C), there was an activity loss of about 18%, which could be attributed to the good stability of BsAbfA during the test. It is obvious that the long half-life and appreciable thermostability of BsAbfA are properties desirable for practical applications.

### 2.4. Kinetic Analysis of BsAbfA

The *K_m_*, *K_cat_,* and *K_cat_*/*K_m_* for *p*NP-α-Af were determined under the optimal conditions for enzymatic reactions catalyzed by BsAbfA and mutants (Table 2). The *K_m_*, *K_cat_,* and *K_cat_*/*K_m_* were 0.6 mM, 108.9 s^−1^, and 181.5 s^−1^ mM^−1^ for BsAbfA, respectively. No activity was detected for E173A and E292A. It is noted that the *K_m_* values of the majority of other mutants, except E173A and E292A, were like those of BsAbfA, whereas the *K_cat_* and *K_cat_*/*K_m_* values of all the mutants decreased significantly. The E173Q mutant had a highest *K_cat_*/*K_m_* that was only 28.8-fold lower than that of BsAbfA, and a *K_cat_* value only 43.6-fold lower. Likewise, the *K_cat_* values of the E173D, E292D, and E292Q mutants decrease by 217.8-, 121.1-, and 68.1-fold, while the *K_cat_*/*K_m_* values decreased by 72.6-, 139.6-, and 78.9-fold, respectively. The *K_m_*, *K_cat_*, and *K_cat_*/*K_m_* values of BsAbfA for ginsenoside Rc were 0.4 mM, 79.1 s^−1^, and 197.8 s^−1^ mM^−1^ (data not listed). The catalytic efficiency of BsAbfA for ginsenoside Rc was higher than that of glycosidase from *S. acidocaldarius* for Rb_1_ (*K_cat_*, 4.8 s^−1^ mM^−1^), Rc (*K_cat_*, 4.5 s^−1^ mM^−1^), Rd (*K_cat_*, 1 s^−1^ mM^−1^), and Rb_2_ (*K_cat_*, 0.8 s^−1^ mM^−1^) [43]. The results suggest that BsAbfA is an efficient enzyme for hydrolyzing ginsenoside Rc.

The effects of metal ions and chemicals on the kinetic parameters of BsAbfA are indicated in Table 3. The BsAbfA activity was obviously enhanced by Mn^2+^, but significantly inhibited by Hg^2+^ and Cu^2+^. The presence of Na^+^, K^+^, Ca^2+^, Mg^2+^, Fe^2+^, Zn^2+^, Ni^2+^, EDTA, DDT, or SDS had no significant effect on the enzyme activity. The results demonstrate that the recombinant BsAbfA had a good catalytic activity and environmental compatibility.

### 2.5. Substrate Specificity of BsAbfA

The substrate specificity of BsAbfA was measured under the optimal conditions using *p*NP-α-Af, *p*NP-α-l-arabinopyranoside (*p*NP-α-Ap), *p*NP-α-l-rhamnopyranoside (*p*NP-α-Rp), *p*NP-β-d-glucopyranoside (*p*NP-β-Glc), Rb_1_, Rb_2_, Rc, Rd, Re, Rg_1_, C-Mc_1_, C-Mc, gentiobiose, and sophorose as substrates. As revealed in Table 4, BsAbfA displayed a high hydrolytic activity on ginsenoside Rc and *p*NP-α-Af, having a preference for Rc. However, BsAbfA had a low activity on C-Mc_1_ and C-Mc, and no activity on *p*NP-α-Ap, *p*NP-α-Rp, *p*NP-β-Glc, Rb_1_, Rb_2_, Rd, Re, Rg_1_, gentiobiose, and sophorose. Of the eight ginsenosides except for Re and Rg_1_, all belonged to the protopanaxadiol-type (PPD-type). In contrast, ginsenoside Rd contains only one glucopyranosyl at C_20_. The main difference between Rb_1_, Rb_2_, and Rc is that the sugar residues substituted at C_20_ of aglycone have sugar moieties (i.e., glucopyranose, arabinopyranose, and arabinofuranose) linking to the glucopyranosyl at C_20_ of aglycone [44]. Owing to the structure at C_20_ like that of Rc, the minor ginsenosides C-Mc_1_ and C-Mc were used as controls to test the specificity. The results suggest that BsAbfA specifically cleaves the outer glucosidic linkage at the C_20_ position of ginsenoside Rc, C-M, and C-M_1_, but does not hydrolyze the inner glucosidic linkage and glucopyranosyl at C_20_ of PPD-type ginsenosides. It also cannot hydrolyze any of the outer or inner sugar moieties, including glucopyranose, arabinopyranose, and rhamnopyranose at the C_3_ or C_6_ (only for the protopanaxatriol-type) position of the ginsenoside skeleton. In addition, the specific activity of BsAbfA for the ginsenosides follows the order of Rc > C-M > C-M_1_. Although the ginsenoside Rc, C-M, and C-M_1_ have the same glycosidic bond at C_20_ position, the recombinant BsAbfA is more active towards Rc and has specific stereo preference for C_3_ sugars. The C_3_ position of ginsenosides C-M and C-M_1_ contains a hydrogen or glucopyranose with small steric structures that may not benefit the formation of hydrogen bonds between Rc and BsAbfA. Therefore, BsAbfA has a high selectivity to ginsenoside Rc, and it hydrolyzes the glucoside at the C_20_ position in ginsenosides, whereas the enzyme does not hydrolyze the glycoside at the C_3_ and C_6_ position. That BsAbfA has the same substrate specificity to the recombinant α-l-arabinofuranosidase CaAraf51 from *C. aquatile* has been previously reported [33].

### 2.6. Biotransformation of Ginsenoside Rc by BsAbfA

**Figure 5:**
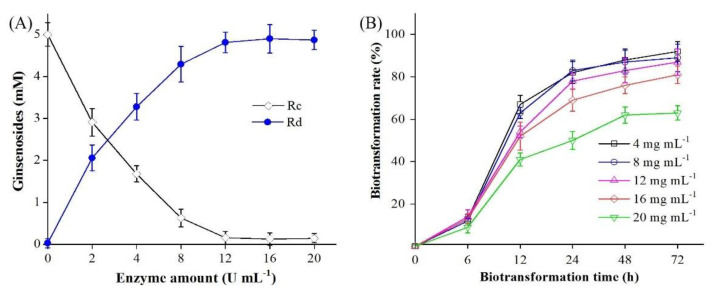
Effect of enzyme amount (**A**) and ginsenoside Rc concentration (**B**) on the production of Rd by using purified BsAbfA. (**A**) The reaction was performed in 50 mM citric acid/sodium citrate buffer (pH 5) containing 5 mM ginsenoside Rc and 0−20 U/mL enzyme at 40 °C for 24 h. (**B**) The reaction was performed in 50 mM citric acid/sodium citrate buffer (pH 5) containing 2−20 mg/mL ginsenoside Rc and 12 U/mL BsAbfA at 40 °C for 0−72 h. The data represent the means of three experiments, and error bars represent standard deviation.

The effect of BsAbfA on the biotransformation of ginsenoside Rc was investigated at pH 5 and 40 °C by varying the enzyme amount from 0 to 20 U/mL enzyme with 2−20 mg/mL Rc for 24 h. As shown in Figure 5A, the molar conversion rate of Rc reached 65% using 4 U/mL enzyme. At 8 U/mL enzyme, ginsenoside Rc was biotransformed to Rd with a corresponding molar conversion rate of 85%. With the increasing of enzyme activity, the productivity of ginsenoside Rd gradually increases, and at BsAbfA of 12 U/mL, the conversion rate of ginsenoside Rc reached the climax and was more than 90%. As shown in Figure 5B, 4−16 mg ginsenoside Rc/mL was efficiently converted to Rd with a more than 80% conversion rate. The conversion rate was still as high as 81% at 16 mg ginsenoside Rd/mL, but decreased obviously above 20 mg ginsenoside Rc/mL. The results indicate that BsAbfA has a good relative stability and high substrate tolerance.

**Figure 6:**
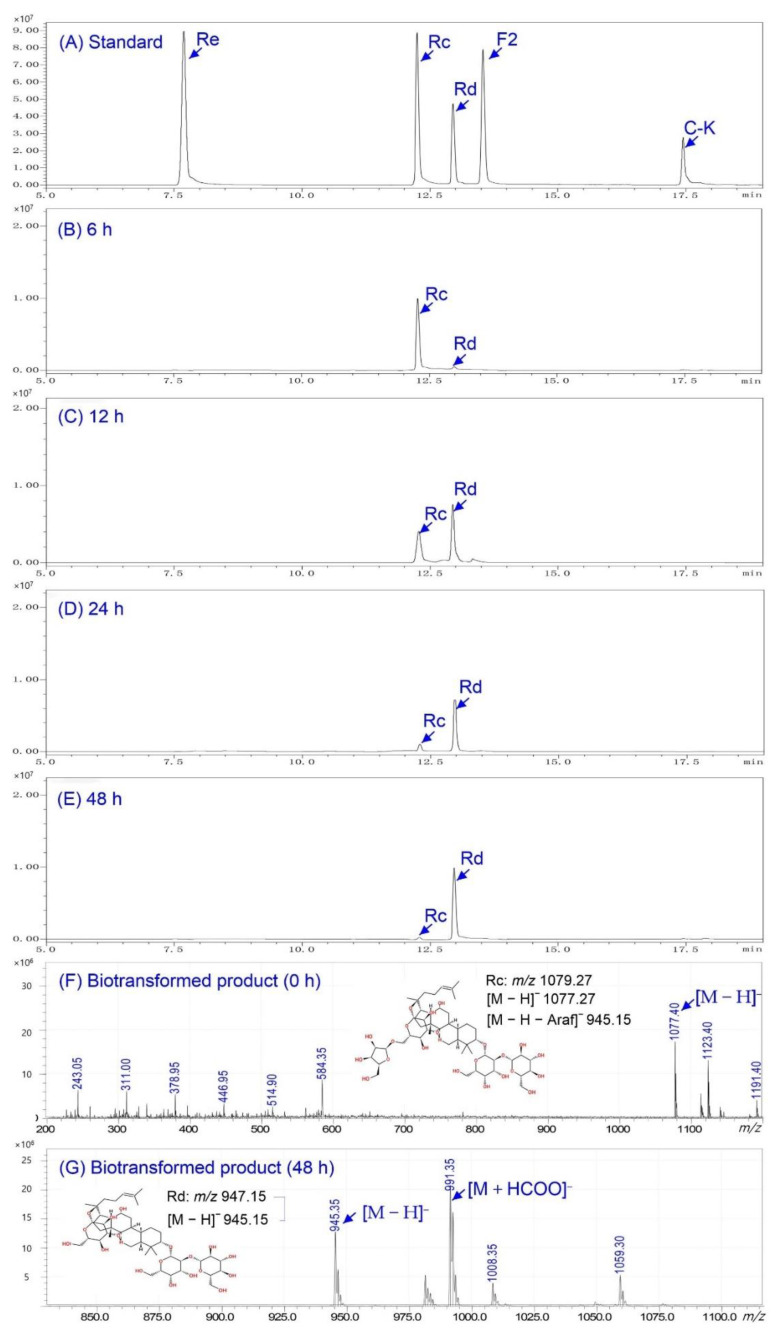
LC/ESI-MS analysis of metabolites of ginsenoside Rc hydrolysis by BsAbfA. (**A**) Standard ginsenoside Rc, Rd, Re, F_2_, and C-K. (**B**–**E**) The biotransformed products of ginsenoside Rc to Rd for 6, 12, 24, and 48 h respectively. (**F**–**G**) Extracted ion chromatograms obtained from the biotransformed products of Rc by BsAbfA for 0 h and 48 h, respectively.

To investigate the transformation mechanism, time-course analysis of the reaction under optimal conditions was performed. The biotransformed products were analyzed by LC-MS and ESI-MS/MS techniques. As shown in Figure 6A–E, there was the detection of ginsenoside Rd using UPLC after 6−48 h, and an obvious decrease of Rc and increase of Rd after 12 h. The results demonstrate that ginsenoside Rc was transformed into Rd in large amounts. At 24 h the Rd yield became much higher and Rd was the sole product of biotransformation. To further verify the transformed products, ESI-MS/MS was used for structural information (Figure 6F). The ESI-MS/MS spectrum of Rc displayed a signal from [M − H]^−^ at *m/z* 1077.27. For the transformed product, there was a signal at *m/z* 945.35 from [M − H]^−^ generated via the loss of HCOOH (46 Da) from [M + HCOO]^−^ at *m/z* 991.35 (Figure 6G). By comparison, the retention time and ESI-MS/MS fragment patterns of the transformed product were the same as those of the ginsenoside Rd standard. Therefore, the sole transformed product was identified as ginsenoside Rd. The BsAbfA exhibited substrate specificity for ginsenosides Rc with arabinofuranose moieties at the C_20_ position, indicating a specific affinity to outer C_20_ arabinofuranose. The proposed pathway of biotransformation ginsenoside Rc by BsAbfA was as illustrated in Figure 2.

### 2.7. Molecular Docking and Examination of BsAbfA Active Site

**Figure 7:**
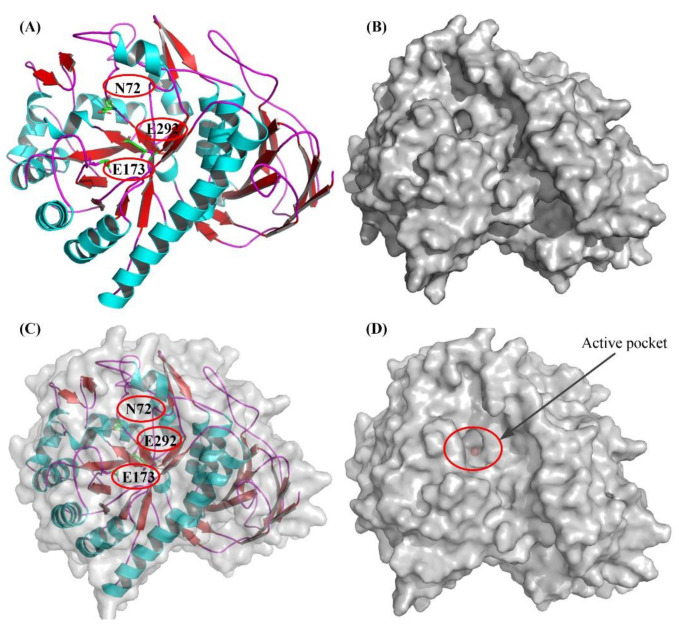
The 3D model of BsAbfA, with location of the deduced catalytic sites indicated. The 3D model of BsAbfA in top view (**A**) and surface view (**B**) was built by comparative modeling using the crystal structure of α-l-arabinofuranosidase Ara51 (PDB code: 5O7Z) from *C. thermocellum* as a template in the SWISS-MODEL server. The colored segment of the skeleton structure marks the position of β-sheet (red), α-helix (cyan), and loop (purple). The red circles show the active pockets or sites. The active sites of three residues (i.e., N72, E173, and E292) are shown as a stick. (**C**) The top and surface view are shown in overlap form. (**D**) The active pocket is shown in a surface view.

From the results of the sequence analysis, it is reasonable to conclude that ginsenosides with arabinofuranose are the likely substrate of BsAbfA. Of the ginsenosides (over 100) that have been discovered, ginsenosides Rc, C-Mc_1_, and C-Mc contain arabinofuranoside bonds at C_20_ of the ginsenoside skeleton. Among them, Rc is higher in abundance, and is often used to prepare its derivatives [16,25,45]. In order to understand the molecular mechanism of the interaction between BsAbfA and ginsenoside Rc, a three-dimensional (3D) model of BsAbfA was built using alpha-l-arabinofuranosidase Ara51 (PDB code: 5O7Z) from *Clostridium thermocellum* [21] as a template in the SWISS-MODEL server, and the sequence identity and similarity were 64.24% and 51%, respectively. To obtain a final structural model, the collected model of BsAbfA was optimized using an energy minimization procedure followed by a short molecular dynamics simulation. As shown in Figure 7, the predicted BsAbfA structure used for docking studies with ginsenoside Rc displayed the outer part of a structural fold that contains α-helices (cyan) and loops (purple), while the inner part is almost all parallel β-sheets (red), forming a catalytically active pocket.

**Figure 8:**
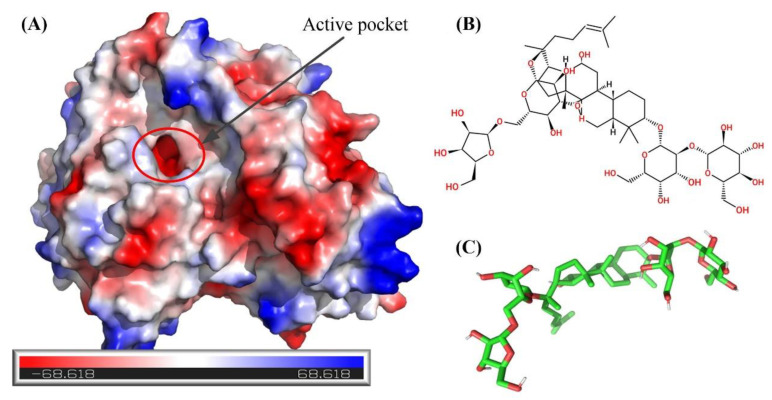
(**A**) Surface electro-static potential plot at the active site of BsAbfA receptor, (**B**) structure of ginsenoside Rc, and (**C**) geometry of ginsenoside Rc (non-polar hydrogens are implicit). Coloring represents the electrostatic surface potential (KbT/ec) with a spectrum of red (electronegative) to blue (electropositive). The red circles show the active pocket.

The surface electro-static potential plot at the active site of BsAbfA receptor (A) and the structure of ginsenoside Rc are shown in Figure 8. The electrostatic surface potential of the model was used to illustrate the potential interactions between the BsAbfA receptor and substrate ginsenoside Rc. As shown in Figure 8A, the electrostatic potential of the hydrolyzed active pocket is a deep negatively charged cavity, suggesting that the glycosidic bond and sugar ring may be attracted as a result of electrostatic interaction, which is one of the reasons for the selectivity of ginsenoside Rc (Figure 8B–C) toward the active pocket.

To understand the structural foundation of BsAbfA hydrolysis, the candidate substrate ginsenoside Rc was docked into the catalytic pocket of BsAbfA. The BsAbfA was held rigid while the substrate ginsenoside was allowed to flex during the docking process. The docking results were analyzed based on the energy and efficiency of polar and non-bonded interactions. It was found that ginsenoside Rc could easily dock into the catalytic pocket of BsAbfA and in different forms. The selected poses of ginsenoside Rc show binding energy from −8.9 to −9.8 kcal/mol, and the dissociation constant was between 6.29 × 10^4^ pM and 2.86 × 10^5^ pM (Table 5).

**Figure 9:**
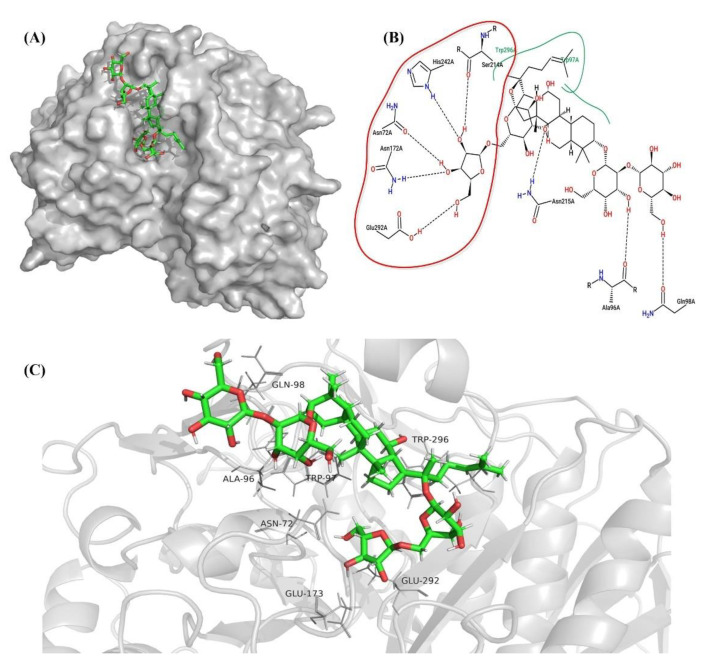
Molecular docking of ginsenoside Rc in the cluster 1 model of BsAbfA (with the minimum docking score). (**A**) Stereoview of the predicted binding of BsAbfA by initial docking. (**B**) Key residues interacting with ginsenoside Rc. (**C**) The binding form and type of key residues with ginsenoside Rc. The dashed line represents hydrogen bonding, and the green line represents hydrophobic interaction.

**Figure 10:**
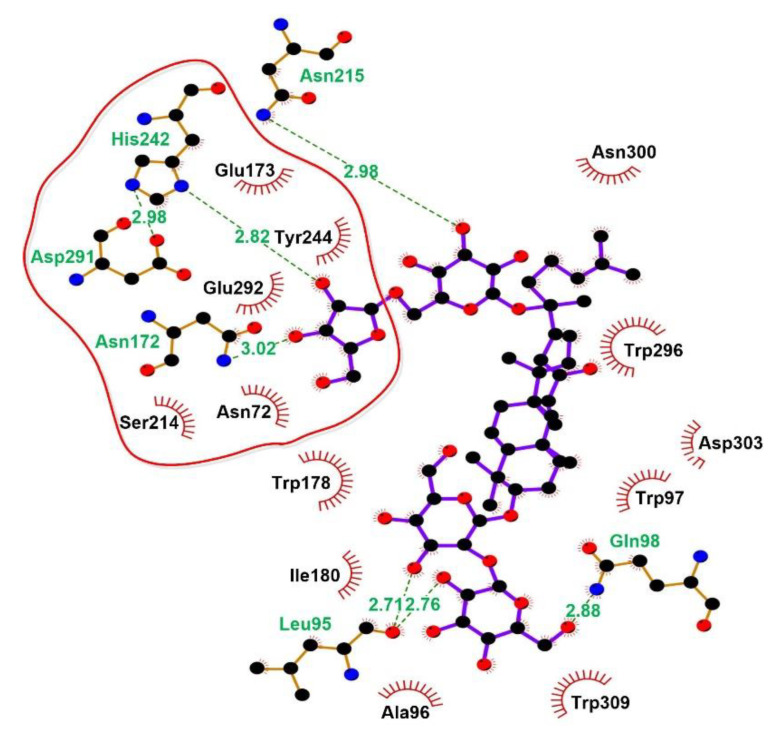
Ligplot output for H-bond and hydrophobic interactions of the amino acid residues of BsAbfA involved in binding with ginsenoside Rc. Hydrogen bonds < 3.1 Å are shown as green dotted lines.

**Figure 11:**
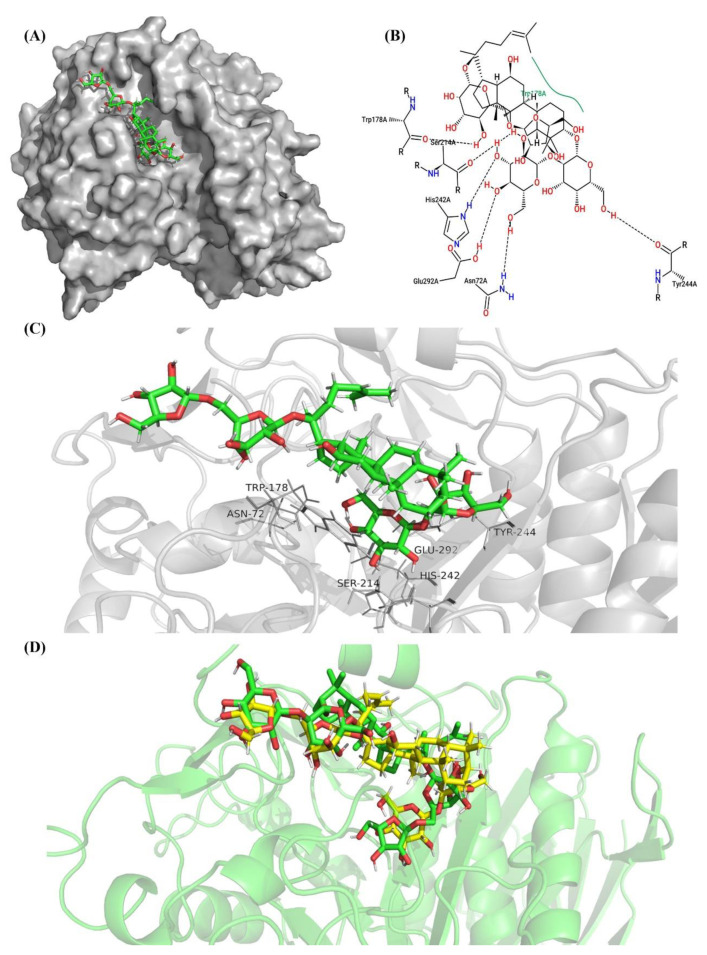
Molecular docking of ginsenoside Rc in the cluster 5 model of BsAbfA (with the maximum docking score). (**A**) Stereoview of the predicted binding of BsAbfA by initial docking. (**B**) Key residues interacting with ginsenoside Rc. (**C**) The binding form and type of key residues with ginsenoside Rc. The dashed line represents hydrogen bond, and the green line represents hydrophobic interaction. (**D**) Docking positions of cluster 5 compared to cluster 1. Ginsenoside Rc represented as yellow (cluster 5) and green (cluster 1) stick model inside a solvent excluded surface (SES) colored by elements.

It is observed that the more negative the docking energy, or the smaller the dissociation constant, the better the binding ability and stability of the ligand with protein. The lowest docking score for the ligand–receptor binding indicates that the binding ability is strong. Therefore, a discussion was conducted based on the minimum and maximum docking score (cluster 1 and 5). The interactions between ginsenoside Rc and binding pocket of BsAbfA are shown in Figure 9, Figure 10 and Figure 11. As shown in Figure 9, ten important amino acid residues, viz. N72, A96, W97, Q98, N172, S214, N215, H242, E292, and W296 of BsAbfA, bind to ginsenoside Rc in the form of hydrogen bond or hydrophobic bond. Among the binding sites, five residues (i.e., N72, N172, S214, H242, and E292) form hydrogen bonds with the outer arabinofuranose moieties at C_20_ position, one residue (i.e., N215) binds to the inner glucopyranose moieties of C_20_ position, especially the hydrogen bond at N72 site is crucial to stabilizing the structure, while E173 and E292 are key residues responsible for substrate catalysis [22,29,30,31]. In this structure, N172 replaces E173 to form the hydrogen bond, while the neighbor residue E173 participates in the hydrophobic interaction with BsAbfA (Figure 10). Furthermore, the hydrophobic interaction of W97 and W296 residues could, relatively, be as important as that of hydrogen bonding. Despite the limit of space in the binding pocket for the accommodation of the fatty acid chain upon C_20_ insertion into the narrow hydrophilic pocket, there is binding of W97 and W296 residues to the fatty acid chain with hydrophobic bonds, squeezing the arabinofuranose into the active pocket as a consequence. There are only two residues (i.e., A96 and Q98) that bind to the glucopyranose moieties at the C_3_ position of ginsenoside Rc, which is at the opposite side of C_20_. Overall, due to the structure of the covalent glycosyl-enzyme intermediate of BsAbfA and ginsenoside Rc, the arabinofuranose cycle at C_20_ easily inserted into the narrow hydrophilic pocket in a stable manner. Accordingly, the C_3_ position of ginsenoside Rc only laid in the groove outside the binding pocket.

Further analysis of the enzyme substrate complexes by ligplot disclosed that one of the best complexes amongst the first 100 contains 19 acid residues that participate to form hydrogen bonds or hydrophobic bonds (Figure 11), and having E173 and E292 in the catalytic cleft or active site. Additionally, the different docking models and experimental results imply that the two conserved E173 and E292 residues are in close contact with the substrate to catalyze the reaction [30,31]. Site directed mutagenesis demonstrated that E173 and E292 mutations of BsAbfA reduce or disable its ability to hydrolyze ginsenoside Rc, indicating that these two amino acids are essential for recognizing hydrolysis of the outer arabinofuranosyl moieties at the C_20_ position. Moreover, the enzyme–substrate docking data showed conformity with the reports concerning similar Glu-based catalytic sites of α-l-arabinofuranose that emphasized the active role of E173, along with the other neighboring amino acids [22,31]. The location and function of these key residues are conservedin GH51 α-L-arabinofuranosidases [30,31], and the result is in good agreement with that of the sequence alignment in Figure 3.

In contrast, if the ginsenoside Rc docked into the BsAbfA in the manner of cluster 5 (Figure 11), there are only seven residues that can bind to ginsenoside Rc, and no bond is formed between the arabinofuranose moiety and BsAbfA. In the BsAbfA complex, the entire ginsenoside Rc can form six hydrogen bonds and one hydrophobic bond in a scattering manner. Therefore, it is less tightly bonded in comparison with the cluster 1 case. Figure 11D shows the two binding forms of ginsenoside Rc and BsAbfA, in which the green and yellow sticks stand for the binding forms of cluster 1 and cluster 5, respectively. The results indicate that arabinofuranosyl is located deeper into the active pocket than glucopyranosyl. Interestingly, according to the *K_cat_/K_m_* of 197.8 s^−1^ mM^−1^, BsAbfA hydrolyzes ginsenoside Rc with higher catalytic affinity. These results suggest that the outer hexose ring at C_20_ position induces a steric hindrance, which makes BsAbfA protein highly selective for the recognition of pentose α-l-arabinofuranose.

## 3. Materials and Methods

### 3.1. Bacterial Strains, Plasmids, and Chemicals

*Escherichia coli* BL21 (DE3) used in this study was purchased from Beinuo Biotech (Shanghai, China). The plasmid pET-28a (+) used as an expression vector was purchased from GenScript (Nanjing, China). The ginsenoside standards of Rb_1_, Rb_2_, Rc, Rd, Re, Rg_1_, F_2_, C-K, C-Mc1, and C-Mc purchased from Chengdu Herbpurify (Chengdu, China) were chromatographic grade. *p*NP-α-Af, pNP-α-Ap, *p*NP-α-Rp, *p*NP-β-Glc, gentiobiose, and sophorose were purchased from Solarbio Science (Beijing, China). All other reagents were analytical grade.

### 3.2. Cloning, Site-directed Mutagenesis, Heterologous Expression, and Protein Purification

The full open reading frame (ORF) of *BsAbfA* gene (GenBank accession: AL009126.3, 2938330-2939832) encoding 1515 bp was synthesized by GenScript (Nanjing, China) after codon optimization [46]. The *BsAbfA* genes separately mutated at E173 and E292 were synthesized by GenScript using a Site-directed Mutagenesis Kit (Nanjing, China). All mutations were confirmed by DNA sequencing. The *E. coli* BL21 harboring *BsAbfA* or mutated gene was cultivated at 200 rpm in Luria–Bertani (LB) medium containing 50 μg/mL kanamycin at 37 °C for 8 h. The cultured bacterium was inoculated to the fresh LB medium to reach OD600 = 0.4–0.6. To induce BsAbfA expression, 0.5 mM IPTG, as a final concentration, was supplemented in LB medium. After the induction, the culture temperature and agitation were reduced to 20 °C and 150 rpm, respectively, and the cells were further incubated for 16 h. The induced cells were harvested by centrifugation (12,000× *g*, 10 min) at 4 °C and stored at 20 °C for further use. Cells were disrupted by sonication with an ultrasonic homogenizer in 50 mM citric acid/sodium citrate buffer (pH 5.5) with 1 g/L lysozyme, as well as EDTA-free protease inhibitor cocktail and 2 mg/L DNase. The debris were removed by centrifugation at 8000× *g* for 20 min at 4 °C. The resulting supernatants were subjected to filtration through a membrane of 0.45 μm. The filtrate was loaded onto Ni-NTA magnetic agarose beads (Qiagen, Germany) for the enrichment of the recombinant BsAbfA protein carrying His-tag. The supernatant was removed by a magnetic separator and washed at least twice with elution buffer. Purified proteins were maintained in 50 mM citric acid/sodium citrate buffer (pH 5) at 4 °C. The concentration of purified recombinant BsAbfA was assayed using Folin-phenol reagent [47]. The expression quantity and molecular weight of the protein were analyzed by SDS-PAGE.

### 3.3. Determination of Kinetic Parameters and Substrate Specificity

In order to determine the enzyme properties of the recombinant BsAbfA, *p*-Nitrophenyl α-l-arabinofuranoside (*p*NP-α-Af) was used as a substrate to assay the enzymatic activities in 50 mM citric acid/sodium citrate buffer (pH 5) at 40 °C. The reaction was ceased by adding 500 mM sodium carbonate with volume equal to that of the reaction. The released *p*-nitrophenol (*p*NP) was immediately measured at 405 nm. One unit (U) of the BsAbfA activity was defined as the amount of enzyme required to generate 1 μmol *p*NP per minute [32]. When ginsenoside Rc was used as a substrate, the reaction was ceased by adding *n*-butanol with a volume 2-fold that of the reaction. The products were assayed by UPLC. The kinetic parameters of BsAbfA were measured using *p*NP-Af and Rc as substrate at concentrations ranging from 0.1 to 5 mM. The *K*_m_*, K_cat_*, and *V*_max_ were calculated by fitting the activity data to a linear regression on Lineweaver–Burk double-reciprocal plots [47]. The substrate specificity of purified recombinant BsAbfA was assayed by using *p*NP-α-Af, *p*NP-α-Ap, *p*NP-α-Rp, *p*NP-β-Glc, ginsenoside Rb_1_, Rb_2_, Rc, Rd, Re, Rg_1_, F_2_, C-K, C-Mc_1_, C-Mc, gentiobiose, and sophorose as substrates, individually. All assays were performed in triplicate.

### 3.4. Effects of pH, Temperature, Metal Ions, and Chemicals on Stability

The effects of pH, temperature, metal ions, and chemicals on BsAbfA activity were investigated using *p*NP-Af as substrate. The buffers used were as follows: citric acid-sodium citrate buffer (50 mM, pH 3–6), sodium phosphate buffer (100 mM, pH 6–8), and glycine-NaOH (50 mM, pH 9–10). The temperature was set ranging from 25 to 70 °C. The relative activity of BsAbfA was defined as 100% at either the optimal pH or optimal temperature. The effects of metal ions and chemicals on the activity of BsAbfA were assessed in the presence of NaCl, KCl, CaCl_2_, MgCl_2_, FeCl_2_, MnCl_2_, ZnCl_2_, NiCl_2_, CuCl_2_, HgCl_2_, EDTA, DDT, and SDS at optimal pH buffer and temperature. All assays were performed in triplicate.

### 3.5. Biotransformation of Ginsenoside Rc

To investigate the catalytic ability of recombinant BsAbfA for the biotransformation of ginsenoside Rc, as well as to disclose the reaction pathway, ginsenoside Rc was dissolved in methanol and incubated in 50 mM citric acid/sodium citrate buffer (pH 5) containing 5 mM Rc and 12 U/mL enzyme at 40 °C. The reaction was sampled at regular intervals for a certain period and ceased by heating the mixture to 80 °C for 15 min. The biotransformed products were subsequently extracted with H_2_O-saturated *n*-butanol. After evaporation of solvents, the products were dissolved in methanol and then subjected to filtration using 0.45 μm microfiltration membranes [48]. UPLC analysis was performed with Shimadzu LC-MS 8050 with a ACQUITY UPLC BEH Shield RP18 column (1.7 μm, 2.1 mm × 50 mm). The mobile phase consisted of acetonitrile (A) and 1% formic acid (B) and the elute program was as follows: A:B (10:90) to (25:75) for 2 min; A:B (25:75) for 2‒8 min; A:B (25:75) to (45:55) for 8‒16.5 min; A:B (45:55) for 16.5‒21.5 min; A:B (45:55) to (98:2) for 21.5‒21.6 min; A:B (98:2) for 21.6‒25 min; A:B (98:2) to (10:90) for 25‒25.1 min A:B (10:90) for 25.1‒29 min. Rc, Rd, Re, F_2_, and C-K were used as standards. The samples were analyzed using a triple quadrupole mass spectrometer equipped with electrospray ionization source. LC-MS analyses were acquired in the negative ion mode by full scan. High purity nitrogen was used as a drying gas, the nebulizing gas flow and heating gas flow were 2 L/min and 10 L/min at 4000 V of ionspray voltage, respectively. The atomizing temperature was 300 °C [49].

### 3.6. Homology Modeling and Molecular Docking

#### 3.6.1. Homology Modeling

The template crystal structure for BsAbfA was identified through BLAST [50] and downloaded from the RCSB Protein Data Bank (PDB code: 5O7Z) [51]. Homology modeling was conducted in the Swiss-model [52]. The target sequence was searched with BLAST against the primary amino acid sequence contained in the SMTL [53]. Models were built based on a target–template alignment using ProMod3 [54]. Coordinates which were conserved between the target and the template were copied from the template to the model. Insertions and deletions were remodeled using a fragment library. Side chains were then rebuilt. Finally, the geometry of the resulting model was regularized by using a force field.

#### 3.6.2. Molecular Docking

AutoDock Vina [55] was employed for molecular docking of BsAbfA with candidate substrate ginsenoside Rc for the prediction of binding affinity and binding sites. The structure of ginsenoside Rc was acquired from PubChem [56] (ID:12855889) and converted to 3D by Avogadro [57] through energy minimization. Then, a conformation search was performed to confirm the stable geometry for the docking preprocessing. As reported in [21], the binding site was located in a beta sheet barrel surrounded by an alpha helix. The three glutamine residues were participants of the hydrolase reaction with a docking box defined at the center of a barrel (50 × 50 × 50 in size). All docked poses of ginsenoside Rc were ranked by binding energy, and the threshold of cluster analysis was set at 5 angstroms. Other parameters not mentioned were set as default.

## 4. Conclusions

The ginsenoside Rc-hydrolyzing α-l-arabinofuranosidase gene *BsAbfA* was cloned from *B. subtilis,* and the optimized recombinant protein was overexpressed and characterized successfully in *E. coli*. The enzymatic properties of BsAbfA are unique and superior to the other reported α-l-arabinofuranosidases, exhibiting higher catalytic efficiency and higher tolerance to metal ions, as well as to organic solvents and detergents. Additionally, BsAbfA shows a high selectivity to cleave the outer arabinofuranosyl moieties at C_20_ of ginsenoside Rc, catalyzing the conversion of ginsenoside Rc to the more pharmacologically active Rd with high productivity. The 3D structure of BsAbfA of family 51, glycosidase modeled by comparative modeling was compact and stable. The docking results revealed that the active site of BsAbfA can accommodate ginsenoside Rc very well. Site-directed mutagenesis of the E173 and E292 residues confirms that it is important to recognize ginsenoside Rc effectively and make it enter the active pocket for the hydrolysis of the outer arabinofuranose moieties at C_20_ position. Thus, we report the BsAbfA as a promising enzyme, and efficient for the industrial production of ginsenoside Rd.

## Figures and Tables

**Table 1:** Production of ginsenoside Rd from ginsenoside Rc by optimized and mutated BsAbfA.

Enzymes	Substrates ^a^	Relative Activity (%) ^b^
BsAbfA	*p*NP-α-Af	100.1 ± 6.2
BsAbfA	Ginsenoside Rc	121.6 ± 4.3
E173A	Ginsenoside Rc	ND ^c^
E173D	Ginsenoside Rc	32.3 ± 2.9
E173Q	Ginsenoside Rc	65.6 ± 2.7
E292A	Ginsenoside Rc	ND
E292D	Ginsenoside Rc	28.7 ± 3.8
E292Q	Ginsenoside Rc	53.4 ± 2.2

^a^ Substrate concentration: 1 mM ginsenoside Rc and 10 mM *p*NP-α-Af. ^b^ The reaction was performed in 50 mM citric acid/sodium citrate buffer (pH 5) at 40 °C for 24 h, and the amount of enzyme was equivalent to 12 U/mL of BsAbfA. The relative activity of *p*NP-α-Af was defined as 100%. ^c^ ND: not detected.

**Table 2:** Enzymatic parameters for hydrolysis of *p*NP-α-Af by optimized and mutated BsAbfA ^a^.

Enzymes	K_m_ (mM)	*K_cat_* (s^−1^)	*K_cat_*/*K_m_* (s^−1^ mM^−1^)
BsAbfA	0.6 ± 0.08	108.9 ± 8.6	181.5 ± 6.9
E173A	ND ^b^	ND	ND
E173D	0.2 ± 0.05	0.5 ± 3.1	2.5 ± 0.06
E173Q	0.4 ± 0.07	2.5 ± 2.1	6.3 ± 0.09
E292A	ND	ND	ND
E292D	0.7 ± 0.06	0.9 ± 0.05	1.3 ± 0.03
E292Q	0.7 ± 0.09	1.6 ± 0.04	2.3 ± 0.05

^a^ The reaction was performed in citric acid/sodium citrate buffer (pH 5), 12 U/mL enzyme at 40 °C for 24 h. The released *p*NP was assayed spectrophotometrically at 405 nm. ^b^ ND: not detected.

**Table 3:** Effects of metal ions and chemicals on the activity of BsAbfA.

Metal Ions or Chemicals	Relative Activity ± SD (%) ^a^	
	1 mM	5 mM
Na^+^	100.4 ± 2.1	98.3 ± 1.7
K^+^	99.8 ± 1.4	93.6 ± 1.5
Ca^2+^	98.4 ± 2.7	93.1 ± 1.9
Mg^2+^	99.6 ± 1.8	91.5 ± 2.3
Fe^2+^	105.7 ± 2.2	95.7 ± 3.1
Mn^2+^	119.7 ± 2.4	109.4 ± 2.5
Zn^2+^	87.1 ± 1.9	77.8 ± 2.4
Ni^2+^	98.8 ± 2.8	91.8 ± 2.5
Cu^2+^	36.8 ± 0.9	24.3 ± 1.1
Hg^2+^	20.3 ± 1.7	5.3 ± 1.1
EDTA	100.2 ± 2.3	99.7 ± 3.1
DTT	98.6 ± 2.7	97.4 ± 2.8
SDS	98.2 ± 2.5	81.2 ± 3.2
Control	100 ± 1.9	100 ± 2.6

^a^ Relative activities of BsAbfA were assayed using 10 mM *p*NP-α-Af as substrate in 50 mM citric acid/sodium citrate buffer (pH 5) with 12 U/mL enzyme at 40 °C for 24 h. The relative activity of *p*NP-α-Af was defined as 100%.

**Table 4:** Substrate specificity of BsAbfA.

Substrates ^a^	Relative Activity (%) ^b^
*p*NP-α-Af	100 ± 3.9
*p*NP-α-Ap	ND ^c^
*p*NP-α-Rp	ND
*p*NP-β-Glc	ND
Ginsenoside Rb_1_	ND
Ginsenoside Rb_2_	ND
Ginsenoside Rc	120.6 ± 2.9
Ginsenoside Rd	ND
Ginsenoside Re	ND
Ginsenoside Rg_1_	ND
Ginsenoside F_2_	ND
C-K	ND
C-Mc_1_	106.2 ± 3.8
C-Mc	108.9 ± 3.5
Gentiobiose	ND
Sophorose	ND

^a^ Substrate concentration: 10 mM *p*NP-α-Af, *p*NP-α-Ap, *p*NP-α-Rp, *p*NP-β-Glc; 1 mM Rb_1_, Rb_2_, Rc, Rd, Re, Rg_1_, F_2_, C-K, C-Mc_1_, C-Mc, gentiobiose, and sophorose. ^b^ The reaction was performed in 50 mM citric acid/sodium citrate buffer (pH 5), 12 U/mL enzyme at 40 °C for 24 h. The relative activity of *p*NP-α-Af was defined as 100%. ^c^ ND: not detected.

**Table 5:** Cluster analysis of the docking position (kcal/mol).

Cluster	Members	Energy	Dissociation Constant (pM)
1	3	−9.824	6.29 × 10^4^
2	2	−9.158	2.19 × 10^5^
3	2	−9.086	2.49 × 10^5^
4	2	−8.991	2.64 × 10^5^
5	1	−8.927	2.86 × 10^5^

## Data Availability

All data supporting the findings of this study are available in the main text.

## Sample Availability

Samples of the compounds are available from the corresponding authors.
